# Emerging Technology for a Green, Sustainable Energy-Promising Materials for Hydrogen Storage, from Nanotubes to Graphene—A Review

**DOI:** 10.3390/ma14102499

**Published:** 2021-05-12

**Authors:** Krzysztof Jastrzębski, Piotr Kula

**Affiliations:** Institute of Materials Science and Engineering, Lodz University of Technology, 1/15 Stefanowskiego St., 90-924 Lodz, Poland; piotr.kula@p.lodz.pl

**Keywords:** hydrogen storage, carbon-based materials, green energy, graphene

## Abstract

The energetic and climate crises should pose a challenge for scientists in finding solutions in the field of renewable, green energy sources. Throughout more than two decades, the search for new opportunities in the energy industry made it possible to observe the potential use of hydrogen as an energy source. One of the greatest challenges faced by scientists for the sake of its use as an energy source is designing safe, usable, reliable, and effective forms of hydrogen storage. Moreover, the manner in which hydrogen is to be stored is closely dependent on the potential use of this source of green energy. In stationary use, the aim is to achieve high volumetric density of the container. However, from the point of view of mobile applications, an extremely important aspect is the storage of hydrogen, using lightweight tanks of relatively high density. That is why, a focus of scientists has been put on the use of carbon-based materials and graphene as a perspective solution in the field of H_2_ storage. This review focuses on the comparison of different methods for hydrogen storage, mainly based on the carbon-based materials and focuses on efficiently using graphene and its different forms to serve a purpose in the future H_2_-based economy.

## 1. Introduction

In our daily life, energy is one of the most integral and necessary elements for us to function. As a result, the constantly growing consumption of energy from various sources is an easy trigger for the emergence of an energy crisis in the area of the whole world. This crisis is related to both the oversupply and demand for energy in every area of life, as well as the constant use of fossil fuels for this purpose. This is mainly due to the constantly growing world population, as well as the growing demand for new technologies and rising living standards in the world. Also, most of the world’s economies are based primarily on the use of non-renewable energy sources [1,2].

It is estimated that up to 80% of the total energy used in the world is the energy obtained from mainly three sources: coal, natural gases, and petroleum. These sources are not inexhaustible, however, the energy crisis related to the use of these fuels may have its climax already in 2035 and last for several years. Limited sources of fossil fuels and rising oil prices, combined with the growing greenhouse gas emissions, pose a challenge to the search for new energy sources and the possibility of using it in many branches of the energy industry [3]. The impact of the automotive industry is particularly important in terms of the excessive use of fuels and the related emission of harmful greenhouse gases and other products of combustion of these fuels into the atmosphere. According to estimations prepared for year 2050, the demand for oil or gas can triple in relation to the current needs [4].

**Figure 1:**
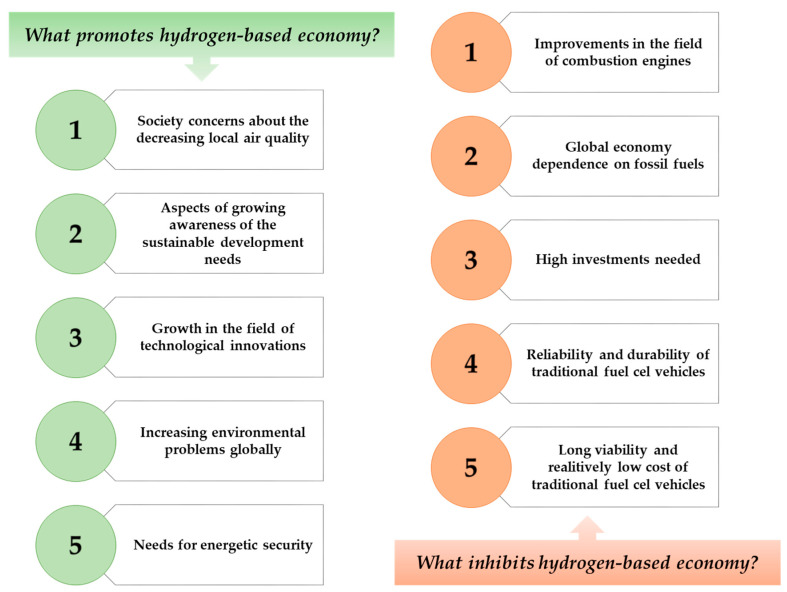
Factors that inhibit and promote the development of hydrogen-based economy.

Hence, by observing the trends in the growing needs of the energy industry, scientists are looking for alternatives to traditional energy sources with the benefit of sustainable methods as an alternative fuel source. On this basis, several years ago, work began to intensify on the use of hydrogen as a sustainable energy source [5,6,7]. On Figure 1, there are presented factors that might promote or inhibit the use of hydrogen as an energy source in not too distant future.

**Figure 2:**
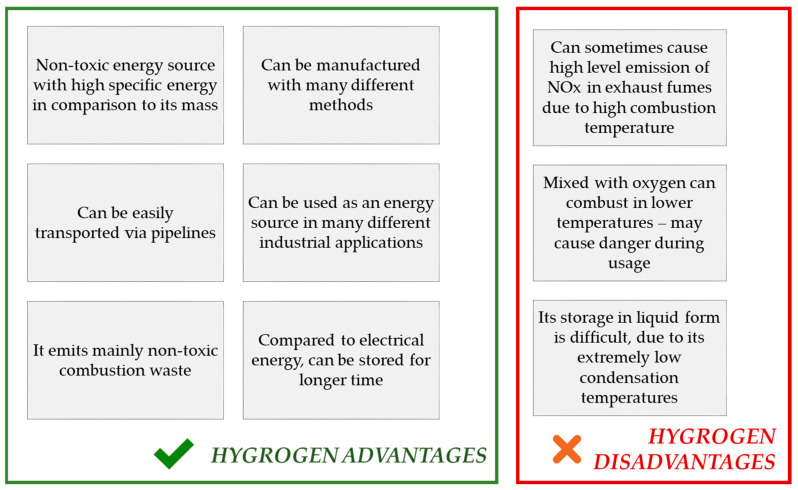
Advantages and disadvantages of H_2_ as an energy source.

Hydrogen is an element of a great importance. As it is the simplest, the lightest, and the most abundant particle on earth, its accessibility makes it a perfect possible solution in terms of many industrial applications, including its use in energy industries. The use of this element has many advantages with very low number of disadvantages, and all these are mentioned in Figure 2.

However, even if hydrogen can be produced via diverse methods, still the requirements on the possibilities of the most effective hydrogen storage has to be exploited. This review examines the possible ways for storing hydrogen as a potential energy source, highlighting the use of graphene-based solutions as a promising perspective in this field.

## 2. Hydrogen Storage—Challenges, Methods, Applications

One of the most difficult challenges for using hydrogen as an energy source is its storage. When designing possible solutions in this area, first of all, one should take into account both economic and purely technological aspects [8].

It can be easily noticed that due to the physical characteristics of hydrogen atoms, the need to find a material for its most efficient storage is a challenging task. The material’s solution for this purpose must fulfill several technical aspects to be considered for further industrial applications. Hydrogen can be stored in three ways [9]:

- Chemically, with the help of different liquid or solid chemical compound;
- Physically (due to the changes of its physical state conditions like pressure, temperature, or phase;
- Or physico-chemically, combining the previous methods.

**Figure 3:**
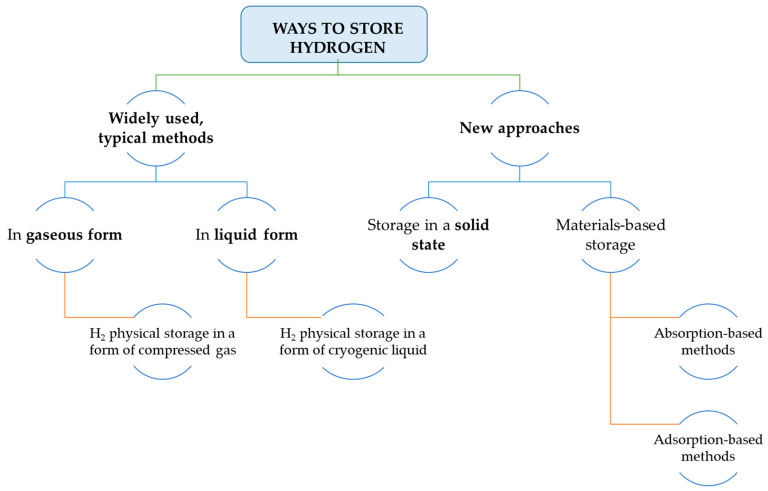
Approaches to store hydrogen (on the basis of [10]).

The division of ways on how to store hydrogen is presented in Figure 3.

A great challenge in hydrogen storing is to allow it to be collected in liquid or solid form. Traditional methods of storing hydrogen, both for stationary and mobile applications, have some limitations, especially due to the very low boiling point (20.2 K) and the extremely low density of hydrogen (0.09 kg/NA m³) in the gaseous and exceptionally high density (70.9 kg/NA m³) in the liquid phase. Additionally, the methods currently used are limited by other factors. First of all, the disadvantage of the methods used is the significant energy loss, which amounts to 40% in the case of condensation and up to 20% of the hydrogen content needed to compress the gas. Moreover, a very important issue is also the necessity to limit the use of high-pressure and cryogenic storage, mainly due to the social aspects that negatively associate the use of gas under pressure and difficulties with stopping the release of liquid H_2_ [11].

**Figure 4:**
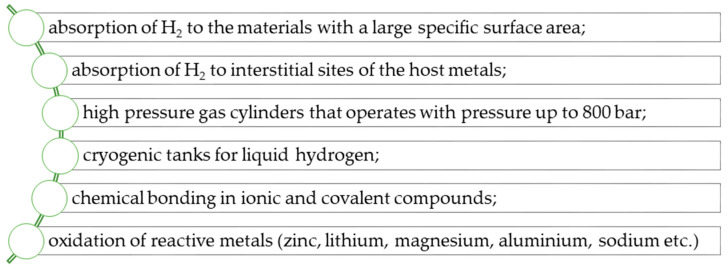
Phenomena and methods used in hydrogen storage methods developed over the past several years (on the basis of [12]).

In recent years, several phenomena and methods has been applied to fulfill the needs of stationary hydrogen storage. Among them, the most popular ones are listed in Figure 4.

A significant technological breakthrough is needed in order to make hydrogen storage efficient and the most economically effective alternative compared to compressed and liquid hydrogen. This means either its solid or liquid storage. However, there is an urgent need to improve the H_2_ absorption and desorption properties in storage materials, mainly due to the fact that its storage in liquid form is difficult because hydrogen condensation requires very low temperatures.

Taking this into consideration, the most promising routes for hydrogen storage are solid materials, whose role will be to chemically bind or physically adsorb hydrogen at bulk densities greater than liquid hydrogen [13].

**Figure 5:**
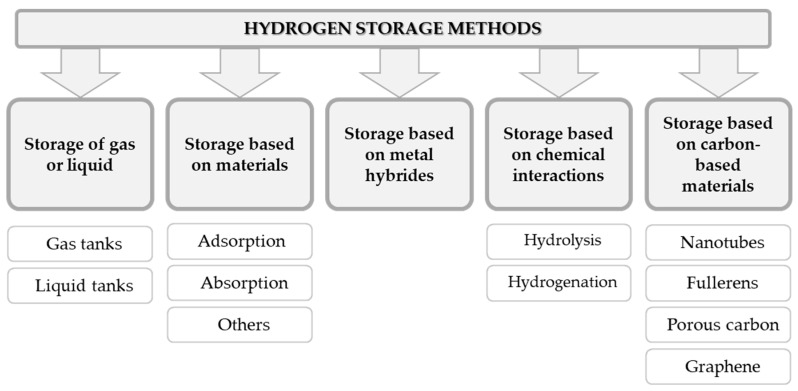
Summary of the most popular hydrogen storage methods [14].

The scheme in Figure 5 presents the most popular methods for hydrogen storage and the mechanisms behind it.

### Industrial Applications for Hydrogen Storage

The literature review of the current state-of-the-art in terms of hydrogen use and the hydrogen storage technologies, points out that in not distant future, the use of H_2_ as an energy source will be present in almost all areas of our everyday life [15].

For now, industrial use of this gas is mostly focused on ammonia production, petroleum, and metals refining [16]. Over these applications, the growing interest in replacing fossil fuels by hydrogen has been intended, mostly in the automotive area.

The need to find an optimized way for hydrogen storage, especially for mobile applications, has originated mostly from the issue of really low density of this gas which results in the need for inadequately large surface area of the fuel tank [17]. In the area of the use of hydrogen as an energy source e.g., in automotive industry, there has been distinguished two ways of performance, one including the rapid combustion of hydrogen in an engine, together with oxygen from the atmosphere, and one in which electrochemically burnt H_2_ and O_2_ (originated from fuel cell) are releasing electrical and heat energy. Both these methods allow the engine to start working, but the problematic issue is not the way how this is supposed to work, but the demanded size for the hydrogen storage area.

Taking these into consideration, it can be clearly seen that storing hydrogen for the mobile purposes still poses a major research challenge for materials engineers. The main difficulties come from the need to achieve a storage medium that enable to store hydrogen with its great density, combined with fast, reversible kinetics of charging and discharging. To achieve this, the considered material requires strong chemical bonds and close atomic packaging. However, to provide a demanded kinetics of adsorption and desorption, requirements are focused on weakly bonded material structures, brittle at moderate temperatures. Moreover, to facilitate a rapid diffusion of hydrogen, loose atomic packaging of storage material and adequate thermal conductivity (in order to prevent heat induced degradation) are needed. By now, scientists seek a way to fulfill all these requirements. From several years, the novel ways developed to serve these purposes come from the field of nanosciences. The possible use of nanosized materials, especially with large surface area hybrid structure, allows the demanded multifunctional action of material serving as the hydrogen storage source [18].

## 3. General Characteristic of Carbon-Based Materials Concerning Hydrogen Storage

The main contraindication to using hydrogen as an energy source in mobile applications is still the low gravimetric density achieved by the available systems (according to the recommendations of the US Department of Energy, it should be 6 wt.% [19]). Carbon in its various forms (e.g., nanotubes, fullerenes, graphene) create a family of substances that enable the storage of large amounts of hydrogen in a reversible manner, which is confirmed by both computer simulations and experimental results.

Graphene belongs to two-dimensional materials, sometimes referred to as single layer materials. It is a class of nanomaterials merely one atom thick. These materials are extracted from layered materials with strong in-plane chemical bonds and weak coupling between the individual layers or artificially synthesized on the substrate. Experimentally, it was first developed via scotch tape peeling of graphite by Andre Geim and Konstantin Novoselov [20]. In its pure form, graphene is a sheet of carbon atoms arranged in hexagon network resulting from sp_2_ hybridization. Material in such form has an obvious tendency to restack and agglomerate. In general, graphite can be treated as numerous layers of graphene that are arranged parallelly via van der Waals bonds that maintain the distance between the planes equal to 0.335 nm [21]. The layers can also be bent into fullerenes or nanotubes. In the first case, the closed ball-like mesh of rings of five to seven atoms originates from carbon atoms that are connected not only via single but also double bonds. In case of nanotubes, the structure is cylindrical like and can be either closed or opened at the ends. That common name refers to single-wall carbon nanotubes (SWCNTs) as well as multi-wall carbon nanotubes (MWCNTs) consisting of nested single-wall carbon nanotubes (CNTs) [22].

Graphene, its properties, and available applications are highly dependent on the manufacturing method used to obtain it. Exfoliation is a process of separation of the layers of the material that can be conducted micromechanically (like during graphene discovery) or in liquid phase. Large-scale production of graphene using this method is rather problematic, which stays against its common use for hydrogen storage. In case of use of chemical vapor deposition (CVD), the manufacturing requires formation of graphene on the substrate due to segregation of carbon atoms dissolved in metals with high solubility of that element [23] or direct nucleation and further expansion on materials with low carbon solubility [24]. Graphene obtained in that manner is of high quality, but similarly to the one obtained by epitaxial growth on SiC [25] or pulsed laser deposition [26], its surface is small. In case of high strength metallurgical graphene (HSMG), the key point of achieving both high quality and large area is the growth of graphene on a liquid metallic matrix [27]. Nevertheless, the obtained material is in the form of sheets that require additional procedures of spatializing [28] to make it applicable for hydrogen storage in, for example, automotive industry. Graphene at mass scale is produced by Hummers’ method based on a multi-chemical treatment of graphite to obtain graphene oxide powders [29]. Probably such form of graphene, after necessary modification, is the most promising for hydrogen storage concerning the low costs of mass production.

The hydrogen storage in carbon-based materials is based both on the physisorption and chemisorption. Physisorption usually occurs with hydrogen in the molecular form and behind its mechanism is the weak interactions with the surface like those based on van der Waals forces, electrostatic and dispersion interactions [30]. The theoretical physisorption of hydrogen on single plane of graphene forming graphite structure is much higher than the value obtained from thermodynamic evaluation of the whole graphite structure. In fact, H_2_ is not able to freely enter between individual planes, and as a result, adsorption occurs mainly at the outer planes [31]. For the most promising results, the distance between individual planes should be enlarged and maintained at the level of about 0.6 nm [32]. From that point of view, the pillared graphene or, in general, spatial carbon-based materials, this group is of higher interest for the sake of hydrogen storage than graphite. The chemisorption requires a chemical reaction between carbon and hydrogen atoms. In the completely saturated state, carbon materials approach 1:1 stoichiometry of C to H. Such state can be achieved for fully hydrogenated graphene that is called graphane.

One of the important aspects of using carbon materials in terms of hydrogen storage is their large specific surface area [33], which has been indicated especially in the case of the use of activated carbon. According to the so-called “Chahine” rule [34], the amount of hydrogen stored can be proportional to the surface development. This means that one of the natural ways of research on hydrogen storage in carbon structures will be associated with the production of nanoporous spatial structures with a large surface, e.g., 3D graphene. Improvement of the gravimetric density of hydrogen stored by systems based on carbon-related materials is also connected with their structural and geometrical modifications or addition of compounds such as metallic catalyst.

The next sections are devoted to the description of various carbonaceous materials potential, starting from those not based on graphene structure, in hydrogen storage. In the part entitled “Graphene-assisted hydrogen storage” the readers will first get acquainted with solutions based on plane graphene and its decoration mostly with metals. The next step is related to the transition from 2D to 3D structures. That can be achieved either via integration of graphene with other materials to form composites or spatialization of graphene itself.

## 4. Hydrogen Storage in Carbon Materials Not Based on Graphene Structures

Fullerenes (for example C_60_), allow to obtain up to 7.7 wt.% hydrogen [35]. However, desorption of this gas takes place at high pressure and temperature (about 773 K and more, 50–120 bar). Fullerenes partially decompose during hydrogen release [36], leading to hydrogen contamination with volatile hydrocarbons [37]. Computer analyses of the decoration of fullerenes with metals show that these materials can reach gravimetric density even above 9 wt.% [38]. Experimental studies conducted with the use of light metals (which include lithium or sodium widely used in these applications) allow to obtain a value of this parameter above 4 wt.%, while ensuring the durability of the deposit. However, they require a hydrogenation temperature of 623 K at a pressure of 100 bar [39].

Both SWCNTs and MWCNTs allow for hydrogen storage in the range of up to 3.5 wt.% [40,41,42] and at pressures up to 140 bar. The maximum degree of nanotube hydrogenation depends on their diameter [43], but the control of this parameter is still a great technological challenge. The advantage of these materials, however, is the room temperature of work. To increase the possibility of hydrogen storage by CNTs, they can be decorated with metals, which has been proved by computer simulations for: titanium (up to 7.7 wt.%) [44], scandium (up to 9.8 wt.%) [45], aluminum (up to 6.15 wt.%) [46], or vanadium (up to 9.2 wt.%) [45]. However, most of the time, the amount of hydrogen stored in decorated CNTs often does not exceed 2 wt.% under experimental conditions [47,48]. However, increasing the gravimetric density of these systems is achieved through the use of low temperatures (e.g., nanotubes decorated with platinum in 125 K made it possible to store about 3.5 wt.% of hydrogen [49]). CNTs decorated with nickel and palladium allow the use of moderate temperatures and near atmospheric pressure, while maintaining high gravimetric densities of the stored hydrogen.

Only 8 wt.% of nickel nano-particles allowed to store 5.27 wt.% of hydrogen obtained as a result of electrochemical reaction and SWCNTs, and 1.35 wt.% in the case of MWCNTs [50]. Mehrabi et al. [51] achieved the release of 2.5 wt.% of hydrogen from MWCNTs containing as much as 25.3 wt.% of nickel and with a temperature above 100 °C. Similar amounts of hydrogen (2.8 wt.%) were stored by MWCNTs with only 6 wt.% of nickel nano-particles, which were produced by the team of Kim et al. [52]. This allowed for the desorption of hydrogen at temperatures from 330 to 520 K. Kaskun et al. [53] presented much worse results for MWCNTs decorated with about 9 wt.% of nickel. The hydrogen content of this material was between 0.114 and 0.298 wt.% at pressures of 4 and 20 bar (room temperature), respectively. Moreover, decorating nanotubes with nickel allows to maintain the hydrogen storage capacity at a similar level in the subsequent cycles of loading and discharging the bed.

**Figure 6:**
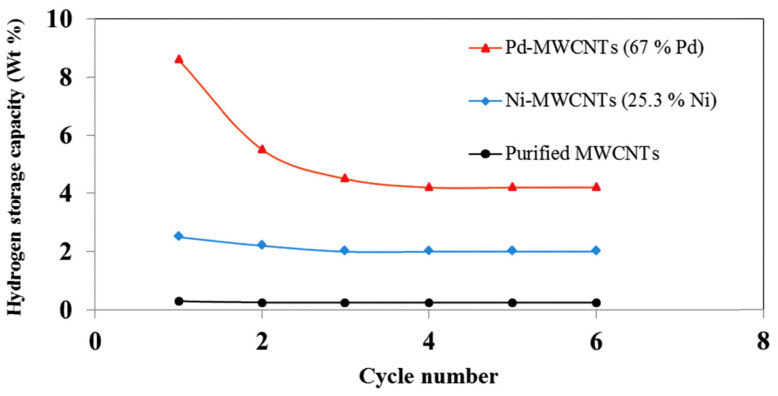
Reduction of gravimetric density of decorated MWCNTs within consecutive cycles of sorption/desorption. Reproduced with permission from Mehrabi et al., International Journal of Hydrogen Energy; published by Elsevier 2018.

On the other hand, decorating MWCNTs with palladium in the amount of 67 wt.% allowed to obtain even 8.6 wt.% of hydrogen at a pressure of 1.5 bar [51]. However, when the hydrogen uptake and release was repeated six times, this value dropped by 54% (see Figure 6). Earlier publications related to the decoration of nanotubes with this metal showed a hydrogen absorption of less than 0.2 wt.% and this is when using higher pressures [54,55].

Not only metals but also their oxides can be effectively used to decorate nanotubes. Vellingiri et al. [56,57] in their publications described the achievement of storing 0.61 to 2.62 wt.% hydrogen in MWCNTs containing up to 9 wt.% SnO_2_ using a pressure of 5 bar and temperature of 373 K. The desorption temperature for these composites ranged from 408 K to 823 K. When using SWCNTs with tungsten oxide, the hydrogen content reaches 2.7 wt.%. The temperature of hydrogen desorption from this material is in the range of 448–578 K. Both in the case of decorating nanotubes with tin oxide and tungsten oxide, the results presented by Silambarasan et al. [58] indicate the possibility of storing only about 1 wt.% of hydrogen at atmospheric pressure and 373 K.

The use of high porosity carbon materials is primarily associated with the use of active carbon. This material can be obtained by treating various types of waste, mainly of plant origin. Obtaining the final product takes place in two stages, in which first the material is subjected to thermal decomposition of organic compounds (carbonization), and then high-temperature modification in an alkaline environment (most often KOH) with the participation of water vapor. The storage of hydrogen in active carbon is probably based solely on the principle of adsorption without the formation of a permanent atomic bond. As a result, the properties of the material under consideration strongly depend on its parameters, such as the size and morphology of the pores, and the specific surface area [59,60]. The commercial use of this type of material is unfortunately limited due to the lack of technology capable of producing pores of a specific geometry and size.

Active carbon [59] obtained by means of biomass carbonization at various temperatures and modification environments, allows to obtain a gravimetric density of adsorbed hydrogen in the range of 3.99 to 5.05 wt.% for a temperature of 77 K and a pressure of 10 bar. This corresponds directly to the specific surface area of the material in the range from 2000 to 3100 m^2^/g and is inversely proportional to the size of the pores.

Similarly, the porous carbon obtained [60] in the processes devoted to the carbonization of rayon fibers allows to develop the specific surface area up to the level of 3144 m^2^/g. For the material obtained in this way, the gravimetric density of adsorbed hydrogen at 77 K and 40 bar pressure ranged from 0.9 to 7.01 wt.%.

The most effective method of modifying the structure of porous carbon is the proper selection of intermediates in such a way as to obtain the desired chemical composition or structure after the hydrocarbonization and activation process. In the work of Blackenship et al. [61], the cellulose acetate used in the proposed work is characterized by a very high oxygen content (oxygen to carbon ratio = 0.83) in the chemical composition, allowing for its concentration in the final product to be controlled and, depending on its value, to analyze the degree of hydrogen sorption. Hydrogen sorbs on such material at a temperature of 77 K in the amount of 3.9 wt.% at a pressure of 1 bar to 8.9 wt.% at a pressure of 30 bar.

Storage of hydrogen in carbon materials such as nanotubes, fullerenes, or porous carbon, for the sake of automotive applications seems to still be a distant future. Conducted experimental researches still mainly result in low level of achieved gravimetric density. Moreover, there are still unwilling handling aspects to overcome such as: instability of those types of materials and their key parameters within the consecutive cycles of sorption/desorption, limitations related to the necessary usage of cryogenic temperatures or high pressures etc. Among the emerging solutions are those based on graphene.

## 5. Graphene-Assisted Hydrogen Storage

For hydrogen storage applications, graphene can be used directly as:

- Basic material that takes part in sorption and desorption processes;
- Scaffolding for metal catalysts allowing the spill-over processes to take place (sorption/desorption of graphene itself assisted by additional catalyst);
- Material used to modify the existing solutions, e.g., composites based on graphene and metal hydrides.

**Figure 7:**
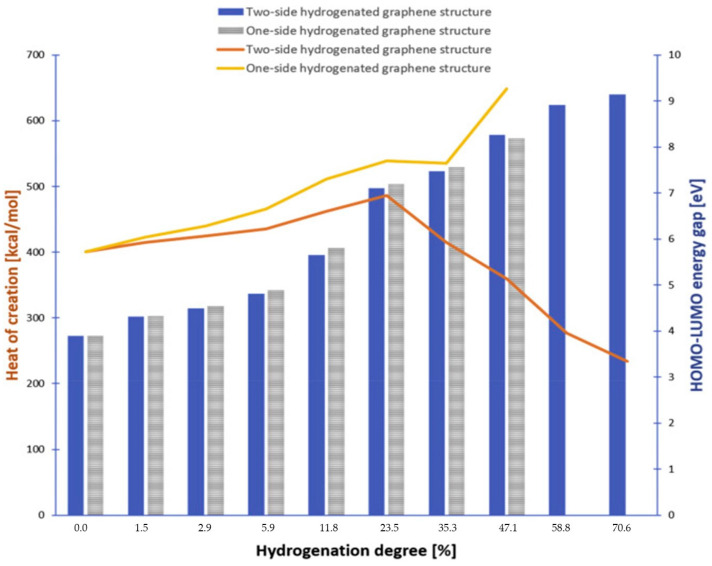
Change in the highest occupied molecular orbital and the lowest unoccupied molecular orbital (HOMO-LUMO) energy gap and the heat of one- or double-sided hydrogenated graphene formation and their hydrogenation degree. Reproduced with permission from Kaczmarek et al., International Journal of Hydrogen Energy; published by Elsevier 2019.

The key to achieving a satisfactory sorption capacity of graphene-based systems is the synergistic effect of hydrogen chemisorption and physi-adsorption. By cyclically treating HSMG graphene sheets [27,62] with the hydrogen plasma action, cyclic changes in electrical resistance were obtained, corresponding to the graphene–graphane transformation [63]. In turn, the molecular modeling method showed that at temperatures interval close to ambient temperatures, hydrogenated graphene–graphane, exhibits hydrogenephilicity in contrast to hydrogenephobic graphene [64]. Figure 7 shows change in thermodynamic stability of graphene hydrogenated to various degrees, based on computational analysis.

### 5.1. Plane Graphene and its Decorated Derivatives

Literature reports indicate that graphene and “graphene” materials in their pure form are not able to exceed the hydrogen storage barrier of a few percent by weight (see Table 1). To achieve this, very high pressure or low temperature are usually used. It should be noted, however, that nowadays reports are usually published covering mostly complex solutions (decorating, multi-component systems, etc.,) and not unmodified graphene. Of course, research is also conducted on the simple forms of graphene, e.g., graphene in the form of nanosheets or exfoliated graphene oxide (GO). The material with the potential to store hydrogen in this form was produced, among others, by Srinivas et. al. [65]. By using only the reduction of GO suspension with hydrazine, they obtained graphene sheets with a specific surface area of 640 m^2^/g. The experimental work carried out at a pressure of 10 bar showed the sorption capacity of this form of graphene at the level of 0.1 wt.% at room temperatures and even 1.2 wt.% for cryogenic temperatures. Kostoglou et al. [66] exfoliated graphene using microwave irradiation to obtain a material with a similar specific surface: about 630 m^2^/g. Such graphene structure at cryogenic temperatures and a pressure of 1 bar was characterized by a sorption capacity of 0.66 wt.%.

In the case of using multilayer graphene as a material for hydrogen storage, the obtained values of sorption capacity depend, inter alia, on the interlayer spacing. During oxidation, it is possible to control the distance between the graphene layers so that the hydrogen storage capacity is as high as possible. This effect can be observed when comparing the sorption capacity of GO and reduced-GO (rGO) by Rajuar et al. [67]. At room temperature, these materials were characterized by H_2_ adsorption of 1.9 wt.% and 1.34 wt.%, respectively, for a pressure of 80 bar and room temperature. The difference was related to the presence of oxygen-containing functional groups that separated the graphene layers more.

**Figure 8:**
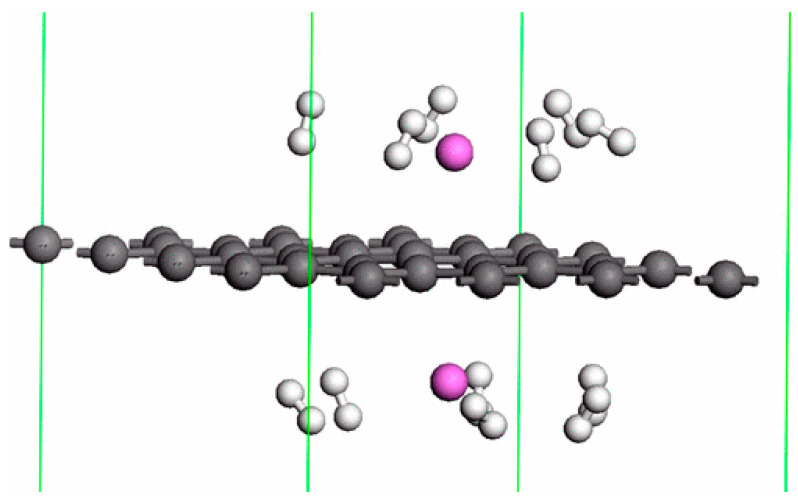
Results of computer simulation of double sided hydrogenated graphene decorated with Al. Hydrogen atoms were marked with white color, while aluminum with magenta. Reproduced with permission from Ao et al., PHYSICAL REVIEW B; published by American Physical Society, 2010.

Many of the published studies in the field of possible use of graphene for hydrogen storage purposes include only calculations or preparation of simulations of possible structures based on this material. These analyses show, that depending on the decorator used, it is possible to achieve not only 5 wt.% when using calcium as a catalyst [79] but even 12 wt.% for lithium [80] or almost 14 wt.% for aluminum [81]. The compilation of computer simulation results for various decorated graphene-based materials are presented in Table 2, while Figure 8 presents the model of Al-decorated graphene prepared by Ao et. al. [81].

The main assumption behind the use of transition metal decorators deposited on graphene structures is related to the so-called “spill-over effect.” This process involves the initial dissociation of hydrogen in molecular form due to the presence of a catalyst dispersed on the surface. Then, migration of hydrogen to adjacent fragments of carbon material is possible, terminated by the diffusion of atomic hydrogen on the carbon support [82,83]. These processes are particularly important in the case of research into materials for storing hydrogen directly at, or very close to, the room temperature. In this way, it is possible to use both physisorption and chemisorption processes to increase the total value of the sorption capacity. Of course, the obtained results will be undeniably influenced by both the fragmentation and uniformity of the catalyst dispersion, as evidenced by numerous publications conducted on various carbon materials, both nanotubes [84] and activated carbon [85].

The mechanism of spill-over processes is a subject of ongoing scientific discussions. There are still voices that its influence on hydrogen storage is exaggerated and the process itself may not take place at all [86]. These voices result, inter alia, from the unsuccessful verification of the research of Li et al. [87] by other research teams [88].

The spill-over effect was confirmed, among many others, by research conducted on the use of platinum as a catalyst. Zhou et al. [89] in his research on this subject used platinum nanoparticles (NPs) immobilized on a graphene composite and zeolitic imidazolate framework (ZIF) thanks to the facile liquid impregnation method. The obtained Pt@ZIF-8/GO material was characterized by a sorption capacity being 2.2 times higher than that of the starting material (ZIF-8) at a pressure of 10.0 bar and a temperature of 298 K.

One of the significant problems in decorating graphene [90] (but also other carbon structures [91]) with transition metals is therefore their agglomeration resulting from high cohesive forces. As a result, the decorated material is heterogeneous, and because of that issue, the uneven distribution of decorators results in a low sorption capacity. Introducing defects to the analyzed graphene structures (both vacancies and additional atoms) helps to eliminate this problem [92,93].

Research conducted on graphene decorated with various concentrations of titanium dioxide (TiO_2_) (from 10 to 15 wt.% of catalyst) [94] showed that both the smaller size of nanoparticles and their large dispersion allow to increase the hydrogen storage capacity by nearly 125%, even despite the reduction of the active specific surface area. The highest obtained final sorption capacity at room temperature and 10 bar, however, did not exceed 0.4 wt.%. In these tests, the decorating effect of graphene was obtained by adding TiCl_3_ to the aqueous GO suspension, followed by drying and thermal reduction processes. The obtained results were significantly higher than in the case of mechanical mixing of TiO_2_ nanoparticles with graphene, probably due to obtaining a better dispersion of the decorator [95]. Hong et al. [96] applied the reverse approach to decorating graphene. In their research, they used a composite made by wrapping graphene around titanium and vanadium oxides. This method consisted of mixing the oxide suspension and then dehydrating them. The obtained structure was stabilized by the presence of hydrogen bonds between the C-OG groups of graphene oxide and the oxygen present in the oxides. The material obtained in this way allowed the H_2_ storage from 1.36 to 1.26 wt.% for GO/V_2_O_5_ and GO/TiO_2_, respectively.

The presence of defects and strictly defined amounts of functional groups (e.g., hydroxyl or carboxyl) also affect the spill-over effect, e.g., by reducing the hydrogen migration energy barriers, which has been confirmed for various carbon materials [97,98] and in particular for nanotubes [54] or activated carbon [99], both in computer simulations and experimental research.

Luo et al. [100] have proposed decorating graphene with scandium, but providing pyridinic-N defects, yielding up to 4H_2_ molecules for each scandium atom in their calculations. Moreover, Kim et al. which worked with the same decorator but doping graphene with boron and obtained about 7.0 wt.% [101].

Research conducted on the doping of B-doped graphene by Faye et al. [102] also confirmed suppressing of metal clustering by the presence of boron. Computer analyses show that the use of copper for the double-sided decoration of such graphene sheets allowed to obtain a gravimetric capacity for hydrogen sorption at the level of 4.2 wt.%.

The purpose of research on hydrogen storage in graphene structures is undoubtedly the broadly understood good of our planet’s humanity and ecology. Interestingly, among the proposed approaches to use graphene for H_2_ storage, you can find those that are also green at the stage of planning the experiment. Vinayan et al. [103] proposed to use the sun to produce a material with potential for hydrogen storage. In this case, the concentrated sunrays took part in the exfoliation of graphene while doping it with nitrogen and reducing the decorator, in this case, lead. The developed material was characterized by a sorption capacity of 4.3 wt.% (at 25 °C, 40 bar).

Nevertheless, these are still simulation values that are unlikely to be obtained in tests. On the other hand, many hopes are attached to various forms of graphene oxide and its reduced form. Moreover, the oxide can be decorated with nanoparticles, just like graphene. The experimental results currently indicate hydrogen adsorption in such structures reaching 5 wt.% [112,113,114,115,116].

**Figure 9:**
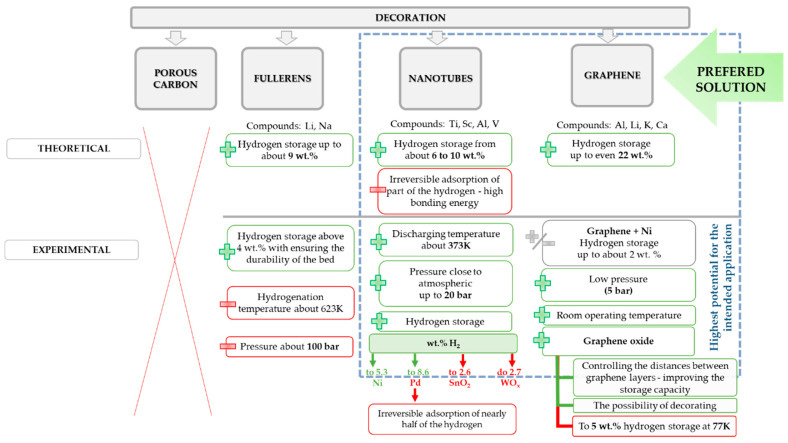
Summary of solutions for carbon-based materials decoration to upgrade their performance for the purpose of hydrogen storage.

The graphical representation of the undertaken approaches of graphene-decoration for the sake of hydrogen storage is presented in Figure 9.

### 5.2. Composites Containing Graphene

Zhang et al. [117] in their work dealt with the LiBH_4_ composite with porous fluorinated graphene structures with a specific surface area of about 220 m^2^/g. As a result of applying 20 wt.% multi-layers sheet-like structures, many important parameters in terms of target hydrogen storage applications have improved. The desorption temperature was lowered by about 120 °C in relation to the unmodified hydride, as well as the kinetics of hydrogen desorption was improved (activation energy decreased from 180.10 kJ/mol to 130.87 kJ/mol, enhanced cycling stability etc.). The developed material achieved a sorption capacity of 3.45 wt.% at 400 °C, which is 2.57 times more than that of unmodified LiBH_4_. The changes resulting from the use of the LiBH_4_ composite and the fluorinated graphene were related to easier recombination of hydrogen molecules on the surface of the material resulting from more reactive sites.

MgH_2_ + NH_4_Cl/graphene composite was proposed by Luo et al. [118] to reduce the temperature of hydrogen release to 437.8 K. This composite was obtained by mixing and grinding all components in a planetary mill in an argon atmosphere for the time period of 3 h. In this case, however, the graphene effect was small reduction of temperature by about 3.5 degree. The improvement of the functional properties by means of magnesium hydride by nearly 463 K was obtained primarily by appropriate selection of protonic and hydric H respectively from NH_4_Cl over MgH_2_. However, the presence of graphene had a positive effect on the purity of the recovered graphene, which was as high as 97.26%. The sorption capacity of the composites was 8.29 wt.% and 7.23 wt.% for the 5% and 10% graphene content, respectively.

Another interesting example of a composite with the potential for hydrogen storage is the combination of magnesium with graphene nanoplatelets (GNPs), i.e., structures containing up to 100 graphene layers [119]. By changing the parameters of the reactive grinding of graphite, from which GNPs with specific properties (size of platelets, specific surface area, etc.,) are obtained, it is possible to accelerate the hydrogen storage kinetics even by an order of magnitude. GNPs improve the migration of hydrogen between nearby Mg particles in various ways. In the case of large GNPs, bridges are formed between adjacent magnesium agglomerates, while small GNPs can create enclaves inside these agglomerates.

### 5.3. Spatial Graphene for Hydrogen Storage

Numerous approaches have been undertaken to move from the plane graphene to its spatial forms: foams or sponges, various scaffolds, pillared graphene, or 3D prints. Even hydrothermal methods can be introduced to achieve spatial forms of graphene [120]. A high-performance self-assembled graphene hydrogel stable in the temperature range between 298 and 373 K containing 2.6 wt.% of graphene enable the electrical conductivity of 5 × 10^−3^ S/cm and in that form can act as supercapacitor electrodes.

In case of spatial graphene manufactured on scaffolds or templates acting as a support, very complex structures can be achieved by means of e.g., chemical vapor deposition or infiltration process in a liquid medium. Chen et. al. [121] produced flexible graphene foam on the nickel and copper support using template-directed chemical vapor deposition process. That structure was stiffened by poly(methyl methacrylate) prior to etching the metal with hot FeCl_3_ solution. For the 3D graphene, the polymer layer was removed with hot acetone. Depending on the type and supportive metal foam the graphene foam with surface area up to 850 m^2^/g and porosity of even 99.7% was achieved.

Ji et al. [122] also used nickel support but with additional thin film of graphitic coating, to achieve N-doped graphene. The spatial structure was manufactured by infiltration of that support with suspension of graphene oxide encapsulated in polypyrole in KOH and ethanol. Additionally, the 3D material was carbonated and activated in 650 °C and the nickel was etched-out. The final specific surface area of that spatial graphene was at the level of 520 m^2^/g. The contamination of the final product with residues of scaffold is a problematic case of methods based on various templates. Moreover, the removal of the metal scaffold is so aggressive that the deformation of desired structures can occur.

Spatial structures with different pore sizes (approximately 1.2 nm, 3.8 nm, and 40–60 nm) resulting from thermal activation in the presence of CO_2_ have been studied by Xia et al. [123]. With the obtained specific surface of the order of 539 m^2^/g, the sorption capacity was achieved for the temperature of 77 K and the pressure of 1 bar, amounting to 0.75 wt.%.

**Figure 10:**
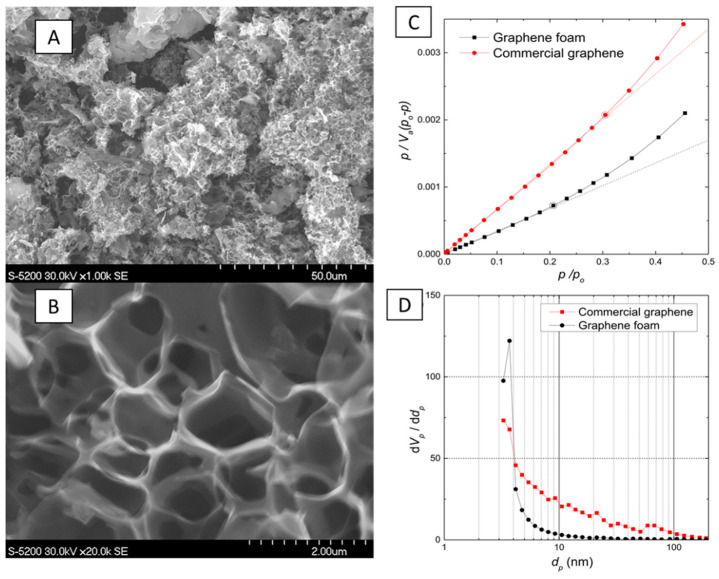
On the left-graphene foam, images obtained via SEM (**A**,**B**). On the right-comparison of commercial graphene and a graphene foam in terms of (**C**) BET surface area; (**D**) pore size distribution. Reproduced with permission from Lyth et al., International Journal of Hydrogen Energy; published by Elsevier, 2014.

Graphene foams with a specific surface area reaching even over 1250 m^2^/g can be obtained for example as a result of combustion of sodium ethoxide. Lyth et al. [124] reported the achievement of 2.1 wt.% of hydrogen storage (77 K and 10 bar) for such surface development. Figure 10 shows both the structure of prepared foam and graphs proving high porosity of that material.

**Figure 11:**
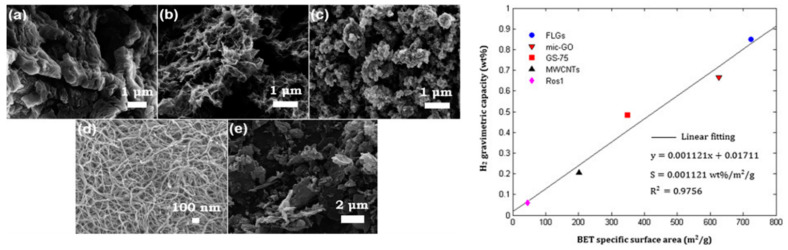
The comparison of nanoporous graphene oxide absorbents. On the left (**a**) exfoliated graphene oxide produced by microwave radiation (mic-GO), (**b**) sponge-like graphene hydrogel prepared by freeze-drying (GS-75)—; (**c**) Commercial few-layer graphenes in the form of nanoplatelets (FLGs); (**d**) commercial multi-walled carbon nanotubes (MWCNTs), and (**e**) commercial short multi-walled carbon nanotubes (Ros1). On the right—lot of correlation between hydrogen gravimetric capacity of these materials and BET specific surface area, measured at 77 K and 1 bar pressure [66]. Reproduced with permission from Kostoglou et al., International Journal of Hydrogen Energy, published by Elsevier, 2015.

Structures known as graphene sponges are obtained, for example, using the technology of freeze drying [66] and a GO suspension treated with hydroiodic acid. As a result of the adopted production method, spatial graphene was obtained with a specific surface area of approximately 350 m^2^/g, which at a temperature of 77 K and a pressure of 1 bar obtained a sorption capacity of 0.85 wt.%. That material was compared with other microwave-exfoliated GOs as depicted in Figure 11.

Spatial porous graphene can be manufactured directly from graphitized coal through multistage processes that involves multiple thermal exfoliation, liquid intercalation, and chemical activation. With such procedure Sun et al. [125] reached the final product with surface area of 2428.6 m^2^/g and pore volume of 1.82 cm^3^g^−1^.

Big family of methods used for the sake of spatial graphene synthesis involves various types of pillars introduced between the graphene planes. Such modification can be based on long-chain polyamides bonding to graphene via amidation [126], polyethyleneimine [127], metal particles [128], or even other carbon-based material as fullerenes [129] or carbon nanotubes [130,131]. Researches conducted by Banda et al. [132] proves that number of pillars has major effect on the properties of spatial graphene. In case of sparsely filling, the gallery structures are preserved, and this facilitates the accessibility to new active sites.

The own research of the authors of this review also focused on a solution with high potential for industrial production of spatial graphene, i.e., using commonly available methods. In their novel 2-step approach, they proposed the use of hydrazine not as a reducing agent but as a compound that cross-links the graphene oxide suspension. This original concept was first confirmed experimentally on a model material-monolayer, quasi-monocrystalline metallurgical graphene HSMG [27], from which a three-layer nitrogen-pillared sandwich was made using local pre-oxidation sites [133]. In industrial scaling, GO was used as a mass substrate. The first step involves the use of an oxygen-containing defects (C=O, –COOH, and –OH groups) substitution reaction with hydrazine carried out at 328 K. As a result, the graphene flakes are pillared with N-N bridges. In the second step, the complete removal of the remaining oxygen groups takes place as a result of reduction conducted in 973 K in hydrogen overpressure. Moreover, the process carried out in this way results in a three-fold increase in the specific surface area (up to 320 m^2^/g) and a ten-fold increase in the pore volume [134]. It should be noted that in these experiments, commercially available graphene suspension was used, however, by reducing the impurities resulting from the production of GO based on the Hammers method, it would be possible to further increase the available surface more appropriately.

Computer simulations on graphene pillared with fullerenes show that at cryo temperatures, the hydrogen uptake of such structures may reach 4.0 wt.% at 1 bar and 10.3 wt.% at 100 bar [135], when the free surface area is over 1755 m^2^/g. In the case of additional decoration of such complex structures with lithium, estimated hydrogen storage capacity may rise to 9.1 wt.% at 77 K and 1 bar [136].

**Figure 12:**
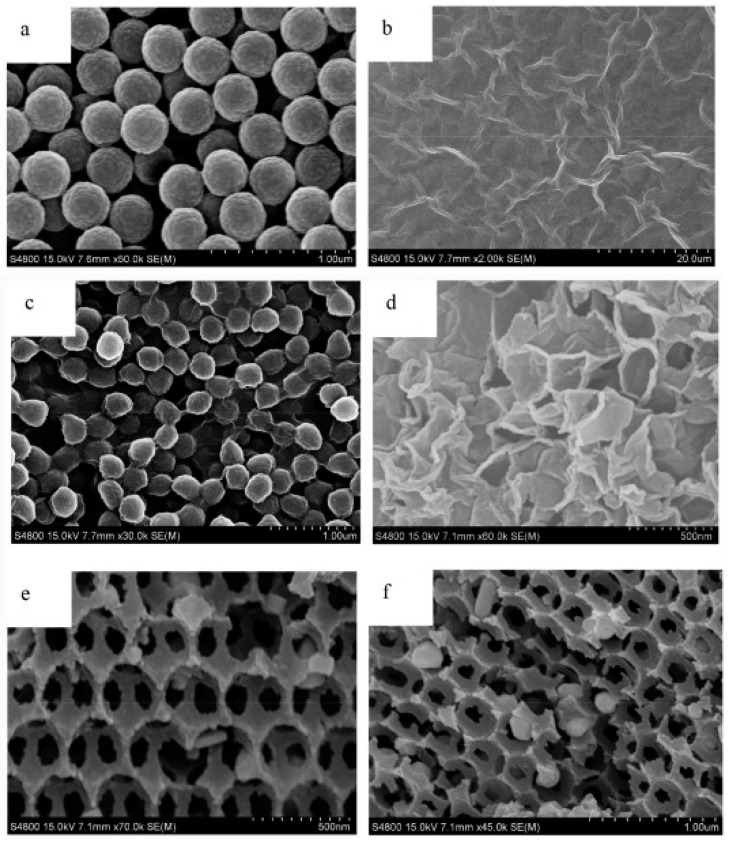
SEM images of (**a**) PMMA microspheres, (**b**) GO-COOH nanosheets, (**c**) PMMA/GO-COOH microspheres assembled through electrostatic interactions, (**d**) porous graphene with nickel nanoparticles-curved layers stack together forming voids (**e**,**f**) 3DHPG-Ni-7.5 nanocomposite-graphene network with pores after pyrolysis. Reproduced with permission from Liu et al., International Journal of Hydrogen Energy; published by Elsevier 2018.

Obviously, the idea of decoration of spatial forms of graphene is under careful investigation. One of the possible approaches involve electrostatic assembling and poly (methyl methacrylate) (PMMA) template [137]. GO-COOH dispersion self-assemble on the surface of positively charged microspheres made of PMMA, and after the addition of NiCl_2_ calcinated in 600 °C in nitrogen atmosphere to remove PMMA. The SEM images depicting stages of composites formation are presented in Figure 12. Such material with Ni nanoparticles located on the graphene porous structures shows hydrogen storage capacity of 1.95 and 4.22 wt.% respectively in 298 K and 77 K under a pressure of 5 bar.

Kostoglou et al. [138] decorated nanoporous carbon structures with a specific surface area of about 780 m^2^/g for which hydrogen sorption was carried out at a pressure of 20 bar. About 2 wt.% of Pt nanoparticles were placed on the surface and in the porosities of this material. At cryogenic temperatures, the selected material performed worse than its undecorated counterpart (sorption decreased by about 7–8%), however, at room temperature, an improvement of nearly 56% was observed. Nevertheless, it should be noted that the highest obtained gravimetric H_2_ uptake for the temperature of 298 K was only 0.131 wt.%.

An attempt was made and described also with HSMG metallurgical graphene decorated with SiC solid particles directly in the phase of nucleation and growth on the surface of liquid copper [28]. The preferential, heterogeneous nucleation of the first and subsequent graphene layers on SiC micro and nanoparticles was demonstrated. These studies were carried out with a view to producing a decorated graphene substrate for the production of spiral, spaced spatial nanostructures [28,139].

Although the amount of hydrogen stored in graphene based materials is still lower than expected, a wide range of investigated solutions (involving different ways of spatialization and pillaring or decoration with various compounds) give hope of upcoming breakthrough in the field of introduction of these materials in green, sustainable energy systems.

## 6. Future Perspectives

Hydrogen as an energy carrier and a potential fuel is a sustainable source of energy, that can be produced from a variety of different sources, both renewable and non-renewable. H_2_ might serve as a promising “fuel of the future” in many different fields of industry, mainly because of its benefits over traditional energy sources in social, economic, and also environmental aspects. Because of that, for the past decades, many investigations have been made primarily to find a way to efficiently and cost-effectively store and transport hydrogen to be used as a potential fuel.

However nowadays, scientists list several technological barriers that need to be removed in order to finish using the carbon-based energy and focus on the systems based on more green resources like hydrogen. Among them, important are the aspects of cost-efficient and sustainable H_2_ production and supply, its storage for not only stationary but also mobile applications, and further costs of hydrogen used as a fuel. All of these aspects directly depend on many economic factors, the development rate of technologies that will be based on hydrogen, as well as the urgent need to reduce the greenhouse gas reduction caused in particular by the extensive use of fossil fuels. Taking these into consideration it must be noticed that the main challenge before making hydrogen a common source of energy is its storage.

**Figure 13:**
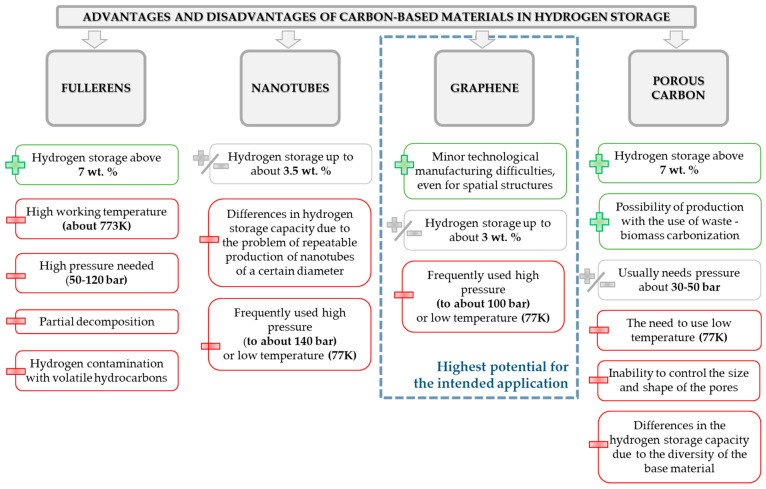
Summary of the advantages and disadvantages of frequently used carbon-based materials intended to use for hydrogen storage.

Over the past years, many various methods have been proposed to fulfill the needs of sufficient H_2_ storage. Among them, we could list methods based on chemical or physical interactions (storage of compressed gas, adsorption/adsorption, use of materials like carbon-based structures). However, the true challenge for development of hydrogen adsorbing materials is to ensure the light-weight, light-carrier materials that have a sufficient amount of bonding sites. Hence, a significant amount of interest of scientists is focused on the use of materials like graphite, zeolites, carbon nanotubes, metal organic frameworks, and many others. The graphical representation of advantages and disadvantages of different carbon-based materials planned to use for hydrogen storage purposes is depicted in the Figure 13.

A significant progress has been made in the development of materials for hydrogen storage of which action will be focused on desorption and absorption properties of nanoscale materials with high specific surface area. Among the proposed solution, one of the promising ways to fulfill the needs of efficient hydrogen storage is the use of graphene-based structures. Authors conclude that spatial graphene decorated not only on the outer surfaces but also inside the porosities, may be a solution for hydrogen storage at ambient temperatures and pressures of at most 5 bars.

The strategic issues for the industrial scaling of hydrogen storage systems based on materials using graphene are:

- Working of the technology of mass production of the substrates—GO and/or rGO with a high degree of exfoliation and purity at the level of at least 99.99% C;
- Development of chemically durable and mechanically resistant, spatially pillared, nanoporous 3D graphene structures with an active surface of at least 1000 m^2^/g;
- Selection of effective “spill-over” catalysts with a range of reversible reactions with hydrogen identical to the reversible reaction of graphene–graphene;
- Development of a technology of spatial decoration of nanoporous 3D graphene structures with nanoparticles of optimal “spill-over” catalysts.

Such graphene-based material may be a key to unlocking the global usage of hydrogen in mobile applications e.g., automotive industry.

## Figures and Tables

**Table 1:** Basic parameters of hydrogen storage with the use of graphene.

Material	H_2_ (wt.%)	Pressure (bar)	Temperature (K)	Remarks
Exfoliated graphene	1.1–1.7	1	77	[65,68,69,70,71,72,73]
	2.15–3	100	293–298	
Graphene	0.9–1.5	100	298	[74,75,76,77,78]
	0.1	10	298	

**Table 2:** Hydrogen storage by decorated graphene—results of computer analyses.

Material	H_2_ wt.%	Remarks
Graphene + Al	13.8	[81]
Graphene + Al	10.5	[104]
Graphene + Si	15	[105]
Graphene + Li	12	[80]
Graphene + Ti	6.3	[93]
Graphene + V	4.6	[106]
Graphene + Ca	7.7	[107]
Graphene + Y	5.8	[108]
Boron-doped graphene + K	22,0	[109]
Boron-doped graphene + Ca	8.0	[110]
Boron-doped graphene + Sc	7.0	[101]
Nitrogen-doped graphene + Pb	4.3	[103]
Ѱ-Graphene + Ti	13.1	[111]

## Data Availability

Data Sharing is not applicable.
